# Modern Medico-Psychology and Psychiatry

**Published:** 1894-06-09

**Authors:** T. S. Clouston

**Affiliations:** Lecturer on Mental Diseases, Edinburgh University, and Physician Superintendent Royal Asylum, Morningside


### Modern Medico-Psychology and Psychiatry.

By T. S. Clouston, M.D., Lecturer on Mental Diseases, Edinburgh University, and Physician Superintendent Royal Asylum, Morningside.

### VII.—THE MEN WHO HAVE MADE MODERN PSYCHIATRY.

In Germany, the greatest name has been Griesinger, of Berlin, who refused to admit that insanity was divorced from other diseases of the brain, and treated and taught it along with other nervous diseases in his wards at the Charité Hospital. That was a great advance, for it broke down all the spiritual and mentally specialised aspect that the disease had acquired before in the eyes of the public and among medical men. Meynert, of Vienna, and Westphal, Griesinger's successor at Berlin, and Gudden, at Munich, made great advances, chiefly on the pathological side, and now there are hosts of workers on the subject in that land of workers, of whom Jolly, of Berlin, and Krafft-Ebing, of Vienna, take the lead. Germany has been more pathological and clinical, and less medically administrative and conditioning, than France or England, though lately there has been erected at Alt-Scherbitz, near Leipzig, an asylum divided up into distinct houses apart from each other, carrying out the most advanced ideas of classifying and conditioning the insane with a view to cure and happiness. Belgium has produced Guislain, and the unique "insane colony" at Gheel, the mother and suggester of all our ideas of domestic treatment and care; Holland, Schroeder Van der Kolk; Switzerland, Forel; Italy, Chiaruggi, Tamburini, Ritti, and Lombroso, all great mental physicians, who have severally advanced our knowledge and added to our means of treating mental diseases.

America has been well to the front in this department of medicine. Rush, Ray, Bell, Howe, Kirkbride, Spitzka, Hammond, Gray, Stearns, Cowles, Folsom, Workman, have been, or are, all most ardent and successful workers. America has been philanthropic, administrative, and also purely medical in her work, and now possesses admirable asylums advancing all the time, with great teaching schools of psychiatry. The asylum at Kankakee, Illinois, consists of a "village street" of houses, and at New York and Boston institutions consisting of many distinct houses are being erected.

In our own country we have not been idle during the century. Prichard was a great physician, of philosophical mind and wide culture, who advanced the science of anthropology as well as wrote the best book up to that time on mental diseases. After Charlesworth, of Lincoln and Gairdner Hill, who advocated the complete removal of mechanical restraint in the treatment of the insane, came Conolly, our fervid apostle of "non-restraint," the eloquent pleader for kindness, mercy, and liberty to the insane; the organiser and medical superintendent of Hanwell Asylum; the most picturesque and interesting figure that our department has yet produced. Conolly was a man of genius, who, after a large and general medical training, took up the cause of the right management of the insane in asylums as Paul took up Christianity, with definite assurance, with fiery zeal, as well as with eloquent and logical argument. He set up a standard of the ideal conditioning of our patients that we are only now attaining in practice. His teaching marked an epoch in the way hospitals for the insane and their inmates were looked on. Before his time county asylums were, by the public, looked on as mere "madhouses" of detention. By all those who read his writings and reports, and they were widely read, the asylum was seen to be a true hospital for cure and philanthropic care. He educated his generation as we all should endeavour by every means to do. After him have come a great crowd of earnest disciples and independent workers. Browne, our Scottish pioneer in this work; Bucknill, the veteran physician, psychologist, author, and asylum superintendent, who still lives, the honoured head of our department in Europe; Skae, my genial predecessor in Edinburgh, whose classification of insanity is the most useful yet devised; Gaskell, Wilkes, Cleaton, and Needham, who, as superintendents of asylums and commissioners, did a great work for these institutions and their inmates. It is invidious to mention those whose swords are still girt and active, but I cannot omit Lockhart Robertson, who, as editor, superintendent, and visitor, has equally distinguished himself; Hack Tuke, whose knowledge, zeal, and industry are equally extensive, having, besides his share with Bucknill in writing their treatise on "Psychological Medicine," written the "History of the Insane," and edited and brought out the most complete account of our subject in the shape of the "Dictionary of Psychological Medicine." Maudsley I have already referred to. Blandford's "Lectures on Insanity" is one of the most practical works ever written on the subject; Crichton Browne, by his zeal and eloquence, by his "Wakefield Reports," by the stimulus he gave to scientific work among his assistants and throughout the English County Asylums, marked an era of psychiatric revival that deserves the highest praise. His successor and greatest pupil, Bevan Lewis, has written unquestionably the most laborious and the most original work in its pathological chapters that has yet appeared in any language. It is truly a monumental work, that will insure immortality to its author. Savage, by his "Manual on Insanity," and his many original contributions to clinical work, and to our knowledge of the pathology of insanity, and by his work as a lecturer at Bethlem Hospital, whereby psychiatric clinical teaching in London has been placed on an absolutely secure footing, has done his share in the modern advance. Mickle has written the most complete work on "General Paralysis" in any language. Sir Arthur Mitchell, the cultivated archæologist, the genial and indefatigable Scotch Commissioner, has made most important contributions to our knowledge of idiocy, but his chief title to future fame will probably be that he was the inventor, promoter, and enthusiastic advocate of a thoroughly organized "boarding out system," whereby between 2,000 and 3,000 persons of quiet habits, though mostly incurable mentally, but capable of increased happiness and enjoyment of life have been mostly removed from asylums and provided with comfortable homes in country cottages, where they live more natural, more domestic, and happier lives under due supervision and guardianship.

